# Missense3D-DB web catalogue: an atom-based analysis and repository of 4M human protein-coding genetic variants

**DOI:** 10.1007/s00439-020-02246-z

**Published:** 2021-01-27

**Authors:** Tarun Khanna, Gordon Hanna, Michael J. E. Sternberg, Alessia David

**Affiliations:** Department of Life Sciences, Centre for Integrative System Biology and Bioinformatics, Imperial College London, London, SW7 2AZ UK

## Abstract

The interpretation of human genetic variation is one of the greatest challenges of modern genetics. New approaches are urgently needed to prioritize variants, especially those that are rare or lack a definitive clinical interpretation. We examined 10,136,597 human missense genetic variants from GnomAD, ClinVar and UniProt. We were able to perform large-scale atom-based mapping and phenotype interpretation of 3,960,015 of these variants onto 18,874 experimental and 84,818 *in house* predicted three-dimensional coordinates of the human proteome. We demonstrate that 14% of amino acid substitutions from the GnomAD database that could be structurally analysed are predicted to affect protein structure (*n* = 568,548, of which 566,439 rare or extremely rare) and may, therefore, have a yet unknown disease-causing effect. The same is true for 19.0% (*n* = 6266) of variants of unknown clinical significance or conflicting interpretation reported in the ClinVar database. The results of the structural analysis are available in the dedicated web catalogue Missense3D-DB (http://missense3d.bc.ic.ac.uk/). For each of the 4 M variants, the results of the structural analysis are presented in a friendly concise format that can be included in clinical genetic reports. A detailed report of the structural analysis is also available for the non-experts in structural biology. Population frequency and predictions from SIFT and PolyPhen are included for a more comprehensive variant interpretation. This is the first large-scale atom-based structural interpretation of human genetic variation and offers geneticists and the biomedical community a new approach to genetic variant interpretation.

**Supplementary Information:**

The online version of this article (10.1007/s00439-020-02246-z) contains supplementary material, which is available to authorized users.

## Introduction

Personalized healthcare, based on an individual’s genetic make-up, is rapidly evolving as a consequence of important technological advances that allow cheap and fast whole-genome sequencing. Examples of the successful implementation of personalized medicine include various types of cancer (e.g. breast and colon), as well as rare diseases, such as cystic fibrosis. However, several challenges remain, including a limited understanding of the genotype–phenotype relationship, which is critical for the identification of clinically actionable genetic variants that can be used in the diagnostic and therapeutic decision-making.

The availability of three-dimensional protein structure data enables one to investigate the effect of genetic variants at the atomic level. The potential of this approach is recognized in the “Best Practice Guidelines for Variant Classification 2020” by the Association for Clinical Genomic Science (ACGS), and is included in the PM1 moderate evidence criteria, deemed able to upgrade evidence for pathogenicity from “moderate” to “strong” in the variant decision-making process (Ellard et al. 2019). However, structural interpretation of human variants remains limited by the relatively small number of experimental structures available for analysis, and difficulties in the interpretation of the results by the biomedical community.

In the absence of experimental structures, three-dimensional structural models of proteins can be accurately obtained using homology modelling. Our *in house* Phyre tool, with over 10,000 citations worldwide, is one of the most used algorithms for this task (Kelley et al. 2015). We recently demonstrated that, with an appropriately parameterized software, three-dimensional models can be used for the interpretation of genetic variants that result in amino acid substitutions, providing predictions with an accuracy approaching that of experimental structures (Ittisoponpisan et al. 2019).

The number of genetic variants in humans is rapidly expanding, but their effect on phenotypes remains unclear. These variants of unknown clinical significance account for a large proportion of the genetic variation annotated in public databases, such as ClinVar (Landrum et al. 2018). New ways to enhance the interpretation of human genetic variants are, therefore, urgently needed and the use of structural data is an exciting new opportunity.

In this paper, we present our work on mapping the entire human proteome to 3D structures using experimental and model coordinates. Moreover, we have calculated the consequences at the atomic level for over 4 million human amino acid substitutions deposited in public databases, including GnomAD (Karczewski et al. 2020), ClinVar (Landrum et al. 2018) and UniProt (UniProt Consortium 2019). These data can be used to prioritize candidate disease-causing variants and identify new actionable human variants. We have made these results available to the scientific community in a dedicated on-line catalogue, named Missense3D-DB (http://missense3d.bc.ic.ac.uk/). A subset of these results, including predictions for variants with a known benign and damaging phenotype, is also freely available as a benchmark resource.

## Methods

FASTA sequences for the human proteome were obtained from UniProt (UniProt Consortium 2019). Human amino acid substitutions were retrieved from the SwissProt Knowledge database (version 2019-06), which includes data from ClinVar (Landrum et al. 2018) and GnomAD (Karczewski et al. 2020). Variants were extracted using their amino acid coordinates. It is, therefore, not possible to exclude that a subset of the variants presented in the database is redundant at the genomic level (e.g. they arise from within or near a tandem repeat). To help the user, we provide the genomic coordinates of each variant (human genome reference builds 37 and 38) in the Results page of the website.

Coordinates for experimental 3D structures were retrieved from Protein Data Bank (PDB) (Burley et al. 2019), whereas 3D coordinates for protein models were built using the Phyre algorithm (Kelley et al. 2015). We deposited the modelled 3D coordinates in our PhyreRisk database (http://phyrerisk.bc.ic.ac.uk/), which is freely accessible to the scientific community (Ofoegbu et al. 2019). Because of the known discrepancy in amino acid sequence numbering between UniProt and PDB, 1:1 mapping was performed for each human sequence using SIFT (Dana et al. 2019). The impact of a variant on a protein structure at the atomic level was performed by assessing the following structural features: breakage of a disulphide bond, hydrogen bond or salt bridge, introduction of a proline or a hydrophilic residue or a charge in buried amino acid, steric clash, charge switch or loss of charge in a buried residue, introduction of a residue with disallowed phi/psi angles, or change in secondary structure. In brief, for each variant, the 3D coordinates of the wild type structure (experimental or modelled) were used to generate a mutant structure (detailed in Ittisoponpisan et al. 2019). The side chain of the target residue (residue to be substituted) and of any residue within 5Å from the target residue, were removed and the side chain of the mutant residue was introduced. Subsequently, the wild-type side chains of the neighbouring residues were reintroduced using SCWRL4, thus generating the 3D coordinates of the mutant structure. The mutant and wild-type structures were compared. Changes that would adversely affect the stability of the mutant structure were predicted when one or more of the above described structural features was present.

For each variant, the structural analysis was performed using 3D coordinates selected based on the following criteria: (1) experimental coordinates were preferred over 3D models; (2) experimental structures were chosen in the following order: (i) X-rays were preferred over NMR and NMR over cryoEM; (ii) structures were divided in bins and within each bin, the structure providing the longest coverage was preferred; (iii) when two structures with the same resolution were available, the structure providing the highest sequence-structure coverage was selected. (3) All 3D models had a query-to-template sequence identity > 30%, a confidence ≥ 95% and length > 30 residues (Ofoegbu et al. 2019). We have made our *in house* pipeline to perform the structural analysis of missense variants (new and already known) available as a free tool for the scientific community (http://www.sbg.bio.ic.ac.uk/~missense3d/) (Ittisoponpisan et al. 2019). Protein disorder (defined as the lack of a single stable tertiary structure) was predicted using DisoPred (Jones and Cozzetto 2015). Only residues forming a intrinsically disordered region, defined by the presence of at least 30 consecutive disordered amino acids (Ittisoponpisan et al. 2017), were considered disordered. The change in free energy (ΔΔG) was calculated using the FoldX software according to its recommended protocol (Schymkowitz et al. 2005) (Delgado et al. 2019). A variant causing a ΔΔG > 1 kcal/mol is considered structurally destabilizing.

For each variant, the following information was collected and is presented in the browser: MAF was obtained from GnomAD (Karczewski et al. 2020) or 1000 Genomes (1000 Genomes Project Consortium et al. 2015), the variant dbSNP Id from the dbSNP database (Sherry et al. 2001), the domain in which the variant occurs was obtained from the Pfam database (El-Gebali et al. 2019) and the Ensembl Id of the transcript harbouring the variant from Ensembl (Yates et al. 2020). Moreover, the conserved coding region (CCR) percentiles and genomic evolutionary rate profiling score (GERP) (Havrilla et al. 2019) were extracted. The Chi-squared test was used to test for enrichment of structurally predicted damaging variants in the most constrained region (CCR ≥ 95 percentile).

For experimental structures, we also determined whether the variant occurs in a residue participating in a protein–protein interaction site by calculating all interacting residues using the biological units of experimental structures of protein complexes deposited in PDB. We considered all biological units without making a selection. Missense variants occurring at protein interaction sites are often damaging (David et al. 2012; David and Sternberg 2015). However, in the current version of the database we do not provide predictions based on the disruption of protein–protein interaction sites. Moreover, for each variant, we calculated in silico predictions, raw score and predicted effect, from SIFT (Sim et al. 2012) and Polyphen2 (Adzhubei et al. 2013). A dedicated database with a web interface was built to present these results, using the python-based framework Flask.

## Results

We extracted and analysed a non-redundant dataset of 10,136,597 human missense variants and mapped 3,960,015 of them onto 18,874 experimental 3D structures and 84,818 3D structural models.

**Fig. 1:**
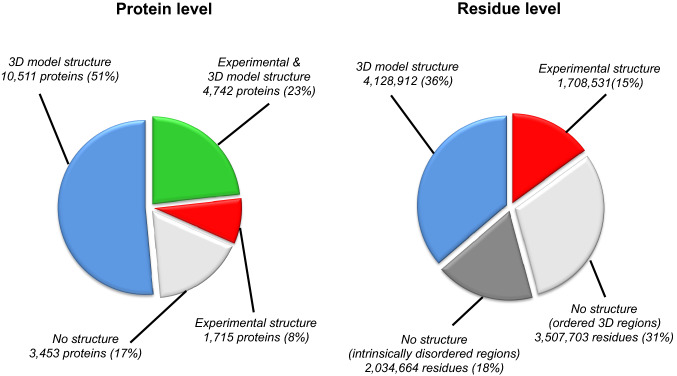
Protein and residue level structural coverage of the human proteome. Coverage was obtained using 3D experimental and model structures

8% (1715 proteins) of the human proteome could be mapped at least partially using only an experimental structure. For 51% (10,511 proteins) of the proteome, no experimental structures were available and coverage was obtained using 3D models. For an additional 23% of the human proteome (4742 proteins), structural coverage could be obtained, using a combination of experimental and 3D model structures. No structural data are currently available for 3453 proteins (17% of the human proteome, Fig. 1).

At residue level, structural coverage is currently available for 5,837,443 (51%) amino acid residues: 1,708,531 (15%) by an experimental structure and the remaining (36%) by a 3D model. 18% (2,034,664) of residues without a structural coverage are predicted to be disordered (Fig. 1).

At variant level, 20% (*n* = 2,437,221) of human amino acid substitutions occur in a disordered region that is predicted to lack a single stable folded structure. Of the remaining 7,699,376 variants, 51.4% (3,960,015 amino acid substitutions) were mapped to an experimental (1,110,833 variants) or modelled (2,849,182) structure and prediction of their effect at atomic level is deposited in Missense3D-DB.

Overall, 14.4% (*n* = 568,548/3,948,327) of missense variants reported in GnomAD are predicted to cause structural damage, thus leading to protein instability or misfolding. Of these 568,548 variants, 2109 are common (MAF > 1%), 377,622 rare (MAF < 1%) and 188,817 extremely rare (no MAF available). Among the missense variants with a known pathogenic annotation, 34.2% (*n* = 6334/18,518) from ClinVar and 36.1% (*n* = 8509/23,588) from UniProt are predicted to cause structural damage. Many missense variants (*n* = 60,354) in ClinVar are annotated as a VUS (variant with unknown significance) or as a variant with conflicting interpretation. Structural analysis shows that 19.0% (*n* = 6266/32,717) of these variants for which 3D analysis could be performed are predicted to cause structural damage.

### The Missense3D-DB web catalogue

**Fig. 2:**
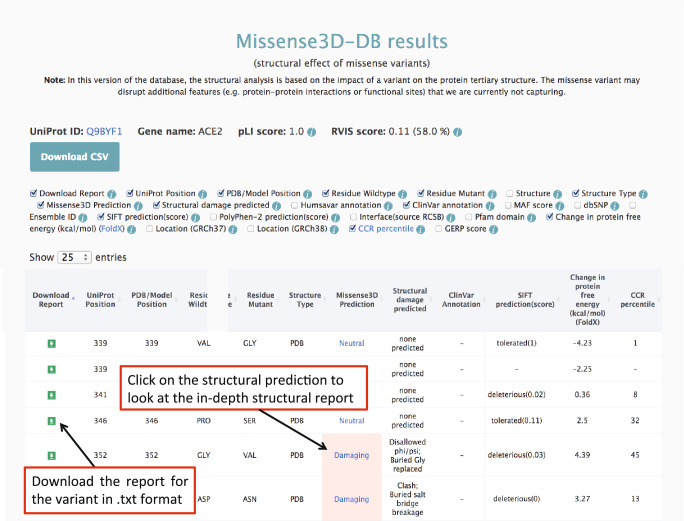
The Missense3D-DB Results page. For each gene, the UniProt Id and gene constrain scores (pLI and RVIS) are reported. For each variant, several annotations can be displayed by clicking the specific annotation icon in the header. A brief description of the annotation can be visualized by mousing over the “i” information icon. The in-depth structural report page can be visualized by clicking on the structural prediction (Neutral or Damaging) of the variant of interest

**Fig. 3:**
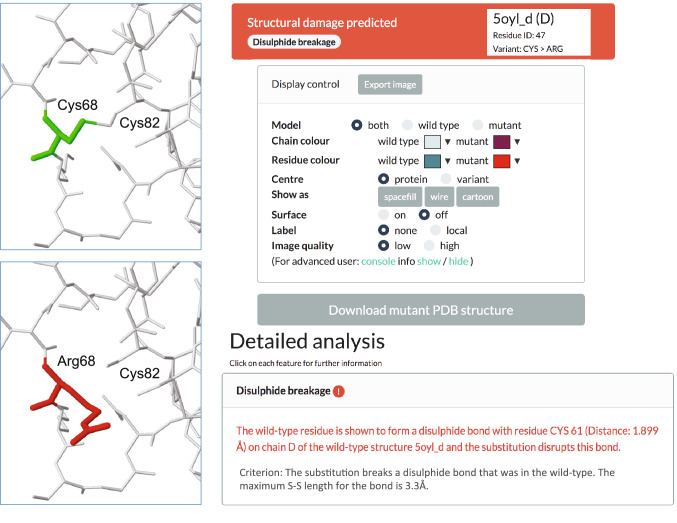
Missense3D-DB: detailed structural analysis for variant p.Cys68Arg in the *LDLR* (NM_000527.4(LDLR):c.202T > C). This variant, annotated in the ClinVar database as “conflicting interpretations of pathogenicity”, disrupts the disulphide bridge between cysteine 68 (corresponding to residue Cys 47 in the 3D experimental structure PDB: 5oyl) and cysteine 82 (corresponding to residue Cys 61 in the same 3D structure)

We designed the Missense3D-DB database and its web interface (http://missense3d.bc.ic.ac.uk/) to enable geneticists and the overall scientific community to access the results of the 3D structural analysis. The browser is free to use and can be interrogated using the gene of interest. For each variant, the Results page reports on the predicted structural effect (benign or damaging), with a concise description of the affected structural features identified by the analysis: e.g. steric clash (Fig. 2). The in-depth structural interpretation is presented in a friendly format for the non-experts in structural biology in a dedicated page accessible from a link in the website Results page. This page contains details on the structural analysis and an interactive viewer that visualizes the 3D coordinates of the wild type and variant structure (Fig. 3). From this site, the 3D coordinates of the variant structure (mutant PDB structure) are available for download.

To provide the user with a comprehensive variant overview, the Results page also reports the Ensembl transcript ID, the domain in which the variant occurs, the variant phenotype annotation according to the database from which it was extracted, the MAF from GnomAD or 1000Genomes, the variant dbSNP ID, and in silico variant effect predictions (including raw score) from SIFT and Polyphen2 (Fig. 2). The changes in folding free energy (ΔΔG) calculated using FoldX are also reported, as well as CCR percentile scores. Although regions with CCR scores ≥ 95% are considered constrained (Havrilla et al. 2019), we did not see enrichment in structurally-predicted damaging variants in these regions. This is likely due to the limitations of our structure-based analysis. As opposed to sequence-based methods, a structural analysis can only be performed when a 3D structure (experimental or modelled) is available and, at present, only ~ 50% of missense variants can be modelled. Moreover, our structural analysis is currently limited to the effect of the variant on the single protein chain and does not take into account additional deleterious effect, such as disruption of protein–ligand and protein–protein interaction sites, and disruption of functional sites (Vihinen 2015).

Results for the query variant, or for all variants mapped for a query gene, can be downloaded from the Results page.

### A set of 31,283 variants for benchmarking new bioinformatics resources

A subset of our results, consisting of 31,283 variants with their structural prediction, is available in the Supplementary Material 1. Variants in this file are non-redundant and annotated as follows: 19,290 pathogenic and 11,993 benign, according to the annotation in ClinVar, Humsavar or both databases. Moreover, variants with MAF ≥ 1% from GnomAD were also included and annotated as benign (Table S1 and Table S2 in the Supplementary Material 2). Variants with conflicting annotation (e.g. benign in ClinVar but pathogenic in Humsavar) were excluded. This resource can be used by the scientific community to benchmark new bioinformatics algorithms.

## Discussion

Large sequencing projects, such as GnomAD (Karczewski et al. 2020), BioBank and the National Heart Lung and Blood Institute Trans-Omics for Precision Medicine (NHLBI TOPMed) Program have released a large number of variants of unknown clinical significance, hence new methods to enhance human genome variation interpretation are urgently needed (Lelieveld et al. 2016; Sevim Bayrak and Itan 2020). Protein 3D structural data can provide important information on the impact of a genetic variant on protein structure and function, thus supporting the decision making on whether the query gene/variant is associated with the disease. Despite the potential of this approach (Ellard et al. 2019; Glusman et al. 2017) its inclusion in variant interpretation workflows has been limited by difficulties in the interpretation of 3D structural data. A survey commissioned by ELIXIR (the intergovernmental European organization established to host life science resources for the scientific community) across the biomedical laboratories of 18 European countries, reported that the difficulty in interpreting 3D structural data is a major barrier to the use of this in variant interpretation. This paper is now available at the following link (10.12688/f1000research.24427.1 in *F1000 research* journal)).

To aid the biomedical community in the use of structural data for variant interpretation, we previously developed a prediction tool, Missense3D, which uses experimental and model structures to predict the structural effect of a missense variant on the protein single chain (Ittisoponpisan et al. 2019). We tested the algorithm on two large datasets of experimental structures and showed that 40% of 1965 missense variants analysed and known to be associated with disease disrupt the 3D structure of the protein in which they occur, as opposed to 11% of 2134 with no known disease-association to-date. Similar results were obtained when the effects of the same variant dataset were investigated using 3D models. These results showed that, in the absence of an experimental structure, a 3D model can be used to derive predictions. Moreover, the relatively low percentage (40%) of disease-causing variants correctly identified is the lower bound of what can be explained structurally. The current version of Missense3D does not take into account additional structural features, such as disruption of interaction or other functional sites. The results of the structural analysis should be considered complementary to those of existing tools based on evolutionary conservation alone (*e.g.,* SIFT) or in combination with the physico-chemical properties of the residue under investigation (e.g, PolyPhen2). These tools have been shown to overestimate damaging variants and often return conflicting interpretations (Miosge et al. 2015; Ittisoponpisan and David 2018). In such cases, 3D structural analysis can help prioritize damaging variants by providing the mechanism by which they affect protein structure and function, as demonstrated on a large study of 12,266 missense variants reported in 641 genes causing endocrine and metabolic disorders (Ittisoponpisan and David 2018).

We have performed the first large-scale, atom-based analysis of human variation occurring in coding regions and have made all results available as an easy-to-interrogate web-based catalogue. Alongside the results of the structural analysis, we provide population frequency and sequence-based predictions from the popular in silico tools SIFT and PolyPhen, for a more inclusive understanding of the variant effect. To further facilitate the interpretation of the results for scientists not familiar with 3D protein structure, we provide a link to an in-depth variant structural analysis page and 3D structure viewer, which mirrors the Results page returned by our Missense3D tool (Ittisoponpisan et al. 2019) (a popular tool for variant interpretation, with > 3000 users since its launch in 2019).

Our atom-based analysis shows that 20% of VUSs that could be mapped onto a structure may be pathogenic, based on their predicted deleterious effect on protein structure. Moreover, a damaging effect is predicted for at least half a million variants reported in GnomAD database. Inclusion of these results in current workflows will enhance variant interpretation and clinical decision-making. Although a missense variant may disrupt protein function by non-structural mechanisms, the 3D analysis can complement and enhance the results obtained from other sources of evidence on gene/disease association. Moreover, the 3D structural interpretation of the effect of a missense variant can provide mechanisms to explain gene/variant pathogenicity that can be tested in vitro.

One limitation of the atom-based approach is that, currently, only 51% of residues in the human proteome can be analysed. However, this percentage is likely to increase significantly in the near future, thanks to world-wide initiatives, such as the Structural Genomics Consortium (Jones et al. 2014), and advances in 3D protein structure modelling, which are expanding the structural coverage of the human proteome.

The current version of the Missense3D-DB database offers a variant interpretation based on the effect on a single protein. The number of damaging variants that we present is, therefore, the lower boundary of what can be structurally predicted as damaging. We previously demonstrated the importance of protein–protein interaction sites as a hotspot for deleterious variants (David et al. 2012) and we are currently working to include predictions of the effect of variants on protein interaction with other macromolecules in the future release of the Missense3D-DB. Because of this limitation in predicting structurally damaging variants, we did not perform an enrichment analysis to ascertain if variants predicted to be structurally damaging are more likely to occur in the constrained area (e.g. those with high CCR or pLi scores).

In conclusion, the atom-based results, freely available in the Missense3D-DB web catalogue, offer geneticists and the biomedical community a new resource for enhancing human genetic variant interpretation.

## Electronic supplementary material

Below is the link to the electronic supplementary material.

Supplementary material 1 (CSV 3023 kb)

Supplementary material 2 (DOC 38 kb)
