# Supplementary material for: Missense3D-DB web catalogue: an atom-based analysis and repository of 4M human protein-coding genetic variants
Source: Hum Genet. 2021 Jan 27;140(5):805–12. doi: 10.1007/s00439-020-02246-z (PMC8052235; doi:10.1007/s00439-020-02246-z)
Supplement: Supplementary file 2 — Supplementary material 2 (DOC 38 kb) [file 439_2020_2246_MOESM2_ESM.doc]

**Missense3D-DB web catalogue: an atom-based analysis and repository of 4M human protein-coding genetic variants**

Tarun Khanna^1^, Gordon Hanna^1^, Michael JE Sternberg^1^, Alessia David^1^

^1^ Centre for Integrative System Biology and Bioinformatics, Department of Life Sciences, Imperial College London, London, SW7 2AZ, UK

**Table S1.** **The set of 31,283 variants for annotation**

| **Source** | **X-rays** | | | **NMR**  **n.** | **EM**  **n.** | **Variants**  **n.** | **Protein**  **n.** |
| --- | --- | --- | --- | --- | --- | --- | --- |
|  | **<2.5Å** | **2.5≤ Å <3.5** | **≥3.5Å** |  |  |  |  |
| Humsavar | 13,903 | 4,382 | 778 | 1,007 | 1,813 | 21,929 | 2,954 |
| ClinVar | 17,893 | 5,957 | 931 | 1,466 | 2,465 | 28,765 | 3,077 |
| GnomAD | 19,688 | 6,436 | 951 | 1,547 | 2,547 | 31,338 | 3,244 |

NMR, Nuclear Magnetic Resonance; EM, Electron Microscopy; **Å**, Angstrom

**Table S2.** **In silico predictions for the** **set of 31,283 variants for annotation.**

Predictions from Polyphen2 could be obtained for 19,094/19,290 damaging and 11,993/11,993 benign variants. Predictions from SIFT could be obtained for 12,609/19,290 damaging and 10,797/11,993 benign variants.

| **Annotation** | **Variants**  **n.** | **Missense3D**  **prediction** | | **Polyphen2**  **prediction** | | **SIFT**  **prediction** | |
| --- | --- | --- | --- | --- | --- | --- | --- |
|  |  | **Damaging** | **Tolerated** | **Damaging** | **Tolerated** | **Damaging** | **Tolerated** |
| **Benign** | 11,993 | 1,768 | 10,225 | 4,274 | 7,719 | 4,384 | 6,413 |
| **Damaging** | 19,290 | 7,337 | 11,953 | 16,528 | 2,566 | 10,799 | 1,810 |

Annotations are from ClinVar or Humsavar. Variants with MAF ≥1% from GnomAD are also included in the benign set.
